# Silibinin improved the function of T cells in peripheral blood mononuclear cells (PBMCs) co-cultured with U-87 MG cell line

**DOI:** 10.22038/AJP.2023.22935

**Published:** 2024

**Authors:** Banafshe Abadi, Jahangir Abdesheikhi, Farnaz Sedghy, Merat Mahmoodi, Hossein Fallah

**Affiliations:** 1 *Herbal and Traditional Medicines Research Center, Kerman University of Medical Sciences, Kerman, Iran*; 2 *Brain Cancer Research Core, Universal Scientific Education and Research Network (USERN), Kerman, Iran*; 3 *Department of Immunology, School of Medicine, Kerman University of Medical Sciences, Kerman, Iran*; 4 *Department of Biochemistry, School of Medicine, Kerman University of Medical Sciences, Kerman, Iran*; † * Equal first author*

**Keywords:** Silibinin, Glioblastoma, IFN-γ, TGF-β, Peripheral blood mononuclear cells (PBMCs)

## Abstract

**Objective:**

Silibinin has exhibited antitumor activities. However, there are few reports about the immunomodulatory properties of silibinin on T lymphocyte function in the tumor microenvironment. Here, we determined the effects of silibinin on T cells of peripheral blood mononuclear cells (PBMCs), cultivated alone or with a human cell line of glioblastoma (U-87 MG).

**Materials and Methods:**

The proliferation of T lymphocytes was assessed by MTT test in the presence of silibinin (15 and 45 µM). Also, total antioxidant capacity (TAC), the activity of superoxide dismutase-3 (SOD3), and the levels of two cytokines interferon gamma (IFN-γ) and tumor growth beta (TGF-β) were compared between treated and untreated PBMCs alone or co-cultured with U-87 cells.

**Results:**

According to our results, silibinin raised the TAC levels and SOD3 activity in the PBMCs and in the co-culture condition. Moreover, silibinin-treated PBMCs showed higher IFN-γ levels and lower TGF-β levels. Interestingly, silibinin protected PBMCs against the U-87-induced suppression.

**Conclusion:**

Altogether, these results proposed the immunomodulatory potential of silibinin on T cells of PBMCs, as well as its partially protective effects on PBMCs against the suppression induced by U-87 MG cells.

## Introduction

Glioblastoma multiform (GBM) is considered one of the most life-threatening forms of brain tumors in adults (Afshari et al., 2019). These patients have a poor prognosis and a survival of (14-15) months from the diagnosis (Hanif et al., 2017). Despite all the advances, GBM remains incurable owing to its invasiveness, disease heterogeneity, and high resistance to chemo-radiotherapy. Therefore, it seems necessary to explore new medicines for glioma treatment (Ferry et al., 2018). Although immunotherapy has recently revolutionized cancer therapy, the entire success has not been achieved in glioma management (Weenink et al., 2020). The main obstacle in treating glioma is the immunosuppressive status of the tumor microenvironment (TME), especially T cells (Woroniecka et al., 2018), which are the most critical arm of anti-cancer immunity. T-cell dysfunction might occur due to the various damaging processes, including continual stimulation/proliferation events resulting in the diminished effector capacity, elevated amounts of tumor-derived reactive oxygen species (ROS) in the environment, T lymphocyte apoptosis, regulatory T cell endurance because of the high quantities of supporting factors like transforming growth factor-β (TGF-β) (Grabowski et al., 2021). 

According to the available evidence, some plant-based compounds show the potential to modify TME (Dias et al., 2021). Silibinin is the most active component of silymarin, obtained from milk thistle (*Silybum marianum* (L.)) seeds (Khalili et al., 2021), and it is widely utilized for treating and preventing hepatobiliary disorders (Abenavoli et al., 2018). Also, this compound possesses several properties, such as anti-inflammatory (Iraji et al., 2022), immunomodulatory, antioxidant, and anti-proliferative abilities (Ranjbar et al., 2020; AbouZid and Ahmed, 2013). In addition, some antitumor capacity of silibinin has recently been described against several types of cancers, such as glioma, breast cancer, and prostate cancer, through different mechanisms (AbouZid and Ahmed, 2013; Jeong et al., 2011; Jung et al., 2009; Jiang et al., 2015; Hosseinabadi et al., 2019; Wu et al., 2009). 

As mentioned earlier, oxidative stress can result in tumor progression and disturb antitumor immunity (Chen et al., 2016). In this context, silibinin sustains the activity of leucocytes by reducing ROS production in the TME. Furthermore, silibinin inhibits signaling of nuclear factor kappa B (NF-κB), a key factor to mediate the expression of tumor-promoting cytokines, such as interleukin 6 (IL-6) and tumor necrosis factor α (TNF-α) (Gao et al., 2015). TGF-β1 is considered a suppressive cytokine for T cells (Jafarzadeh et al., 2017), mainly by impairing interleukin 2 (IL-2) secretion and inhibiting the cytotoxic T cells (CTLs) function through declining the levels of interferon-γ (IFN-γ), perforins, granzymes, and Fas ligand (Thomas and Massague, 2005). 

As the immunomodulatory effects of silibinin in glioblastoma have not been fully understood, we examined the effects of silibinin on the viability and cytokine production of T cells in peripheral blood mononuclear cells (PBMC), alone or co-cultured with a human cell line of glioblastoma (U-87 MG), to determine the potential of silibinin to modify the TME. 

## Materials and Methods

**Ethical statement**

This study was performed after approval of the Ethics Committee of Kerman University of Medical Sciences (98001100). All participants signed informed consent before enrollment.

**PBMC isolation**

PBMCs were isolated from the blood of 5 healthy volunteers. PBMC separation was performed using density gradient centrifugation in Ficoll-Paque medium (Sigma, USA). In summary, after 1:1 dilution of whole blood with phosphate-buffered saline (PBS), samples were added into a conical tube containing Ficoll-Paque solution and were centrifugated at 800 x g for 20 min at 25°C. After centrifugation, the PBMC layer was immediately washed with PBS. The cells were then resuspended in complete Roswell Park Memorial Institute (RPMI)-1640 medium (Capricorn Scientific, Germany) containing 10% heat-inactivated fetal bovine serum (FBS) and 100 U/ml penicillin plus 100 μg/ml streptomycin (Gibco, Germany). Ultimately, they were incubated under standard conditions (at 37°C, 5% CO_2_, and 95% humidity). 

**Cell line**

The human glioblastoma U-87 MG cell line was purchased from the national cell bank of Iran (Pasteur Institute, Tehran, Iran). The cells were cultivated in complete RPMI-1640 medium, at 37°C, 5% CO_2_, and 95% humidity. When reaching approximately 80% confluency, the cells were cultured into flat-bottomed adherent 48-well plates. 

**PBMC proliferation assay**

The proliferation of PBMCs was investigated using MTT [3-(4,5- dimethylthiazol-2-yl)-2,5-diphenyltetrazolium bromide] (Sigma Aldrich, USA) assay. For this purpose, 7×10^5^ PBMCs were cultivated alone or with 7×10^4^ U-87 cells (Effector:Target ratio, 10:1) in a 48-well plate. Anti-CD3 (Biolegend, clone OKT3) and anti-CD28 (Biolegend, clone CD28.2) antibodies were used to stimulate T cells in PBMCs. Silibinin (Sigma-Aldrich, USA) (0, 5, 15, 45, and 135 µM) were then added to cells. After 72 hr, MTT dye at 0.5 mg/ml concentration was added to all the wells and incubated for 3 additional hours. Afterward, the media were removed, and dimethyl sulfoxide (DMSO) (Merck, Germany) was used to dissolve the formed crystals. Finally, the absorbance of the wells was measured at 570 nm and the %protection of silibinin on PBMCs was calculated using the following formula:

$\mathrm{\%Protection}=\frac{\mathrm{ODTcc}-\mathrm{ODTp}}{\mathrm{ODCcc}-\mathrm{ODCp}}$

ODTcc= absorbance of PBMCs co-cultured with U-87 MG cells in the treated condition

ODTp= absorbance of PBMCs alone in the treated condition

ODCcc= absorbance of PBMCs co-cultured with U-87 MG cell line in the non-treated condition

ODCp= absorbance of PBMCs alone in the non-treated condition

**Determination of U-87 MG cell proliferation**

MTT test was used to measure the proliferation rate of U-87 MG cells. 7×10^4^ U-87 cells were cultured in a 48-well plate, exposed to silibinin (0, 5, 15, and 45 µM) and incubated in above-mentioned conditions. MTT dye at 0.5 mg/ml concentration was added and after 3 hours, the media were removed, and DMSO was added to the all wells.

**Determination of antioxidant** **parameters**

Total Antioxidant Capacity (TAC) and superoxide dismutase 3 (SOD3) activity were compared between the supernatant of treated and non-treated PBMCs. TAC was determined based on the ferric-reducing antioxidant power assay (FRAP) method (ZellBio GmbH, Germany). The results are expressed as μmol/L and U/ml for TAC and SOD3 activity, respectively. 

**Cytokine assay**

An ELISA method (Karmania Pars Gene, Iran) was used to determine the amount of IFN-ɣ and TGF-β from PBMCs cultured alone or with U-87 cells and compared between the silibinin-treated and non-treated conditions. 

**Statistical analysis**

Data were statistically analyzed using GraphPad Prism® 6.0 (San Diego, CA). The conformity of data distribution in experimental groups was assessed by the Shapiro-Wilk test. One-way analysis of variance (ANOVA) (or nonparametric equivalent) test followed by Tuckey's post hoc test was applied to compare the levels of variables between groups. The data are presented as the mean±SD (standard deviation). Differences with a p-value <0.05 were considered statistically significant. 

## Results

**Silibinin protected PBMCs against the suppressive potential of U-87 MG cell line**

**Figure 1:**
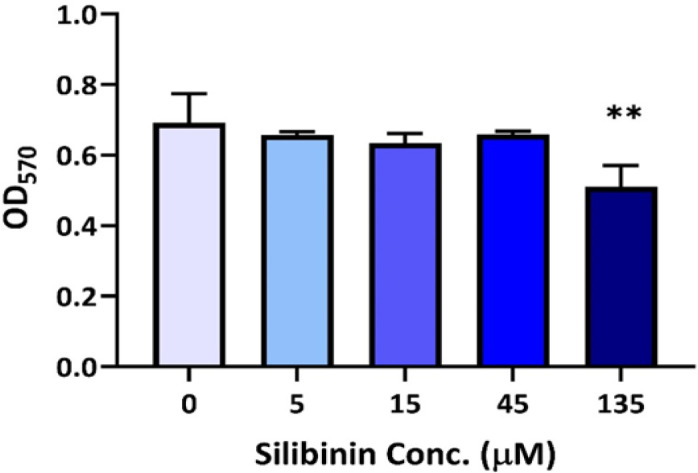
The effect of silibinin on the proliferation of T cells in PBMCs. The effect of four concentrations of silibinin was determined on the T-cell proliferation (n=3). The graphs illustrate the mean±SD. Asterisks show significant changes compared to non-treated cells (**p<0.01).

**Figure 2:**
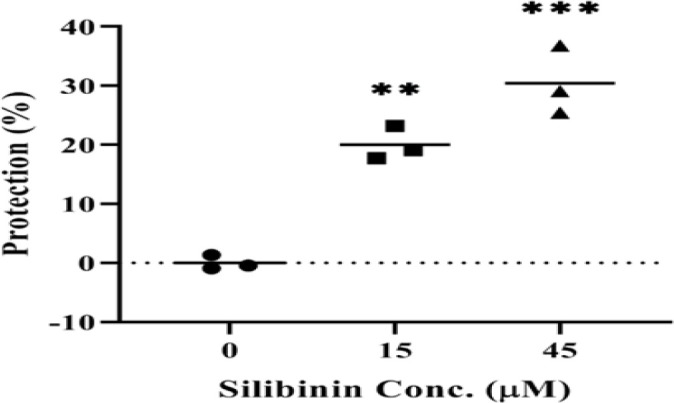
Silibinin protection on T cells co-cultured with U-87 MG cell line. The protective effect of two silibinin concentrations was assessed on T cells in PBMCs cultured with U-87 MG cells (n=3). The graphs illustrate the mean±SD. Asterisks shows significant changes as compared to non-treated cells (**p<0.01 and ***p<0.001).

PBMCs were exposed to silibinin and cultivated with or without U-87 MG cells for 72 hr at 37°C, 95% humidity, and 5% CO_2_. Anti-CD3 and anti-CD28 antibodies were used to elicit the proliferation of T cells. MTT results illustrated that silibinin did not have any negative impact on the proliferation, but a significant decrease was observed in T-cell proliferation with 135 µM silibinin (p=0.0029, Figure 1). Also, there was a substantial protective effect of silibinin (20% protection, p=0.0012 with 15 µM; and 30.45% protection, p=0.0001 with 45 µM silibinin) on PBMCs against the U-87-induced suppression (Figure 2). 

**Silibinin had no effect on the** **proliferation of U-87 MG cell line**

**Figure 3:**
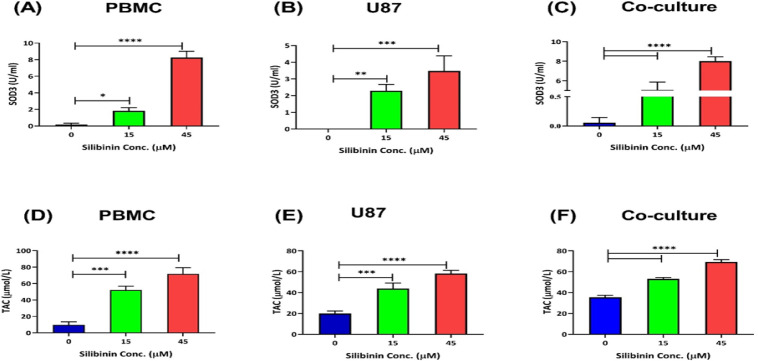
Silibinin effect on U-87 MG cells. The effect of two concentrations of silibinin was assessed on U-87 MG cells (n=3). The graphs illustrate the mean±SD.

U-87 MG cells were cultivated with silibinin (0, 15, and 45 µM) and cultured for 72 hr. MTT results indicated no significant change in proliferation of U-87 MG cells treated with different concentrations of silibinin (p>0.05, Figure 3).

**Silibinin enhanced antioxidant activity in PBMCs alone and in co-culture with U-87 cells**

**Figure 4:**
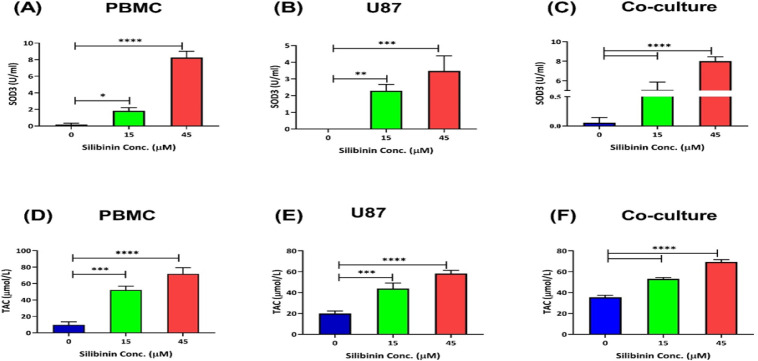
Silibinin effect on the oxidative activity of T cells, U-87 cells, and coculture condition. Silibinin effect on the SOD3 activity and TAC levels was compared between PBMCs (A and D), U-87 MG cells (B and E), and in the co-culture condition (C and F) (n=3). The graphs illustrate the mean±SD. Asterisks show significant changes as compared to non-treated cells (*p<0.05; **p<0.01; ***p<0.001; and ****p<0.0001).

The anti-oxidative capacity was determined in PBMCs and in the co-culture in the presence of silibinin. PBMCs were treated with silibinin (15 and 45 µM), and anti-CD3 and anti-CD28 antibodies were added. We found higher SOD3 activity (p=0.0126 with 15 µM and p<0.0001 with 45 µM; Figure 4a), and TAC in treated PBMCs, especially with 45 µM silibinin (p=0.0002 with 15 µM, and p<0.0001 with 45 µM; Figure 4d). Also, SOD3 activity and TAC were higher in the co-culture condition after treatment with both concentrations of silibinin (Figure 4c and 4f, p<0.0001). 

**Silibinin enhanced IFN-γ levels in PBMCs**

**Figure 5:**
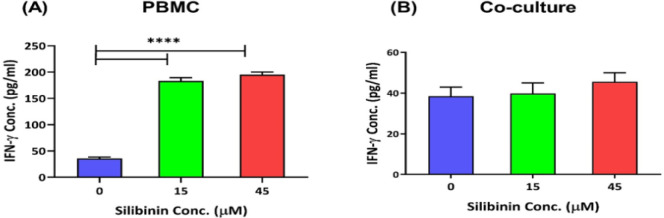
The effect of silibinin on the IFN-γ levels of T cells in PBMCs and in the coculture condition. The effect of silibinin was determined on the levels of IFN-γ in T cells of PBMCs (A), and in co-culture condition (B) (n=4). The graphs illustrate the mean±SD. Asterisks show significant changes as compared to non-treated cells (****p<0.0001).

The IFN-γ release was examined in PBMCs and in the co-culture after treatment with two concentrations of silibinin. According to our data, both concentrations of silibinin significantly enhanced IFN-γ levels in PBMCs (p<0.0001, Figure 5a). However, IFN-γ secretion was similar in the co-culture condition when comparing treated and untreated cells (Figure 5b). 

**Silibinin reduced TGF-β levels in PBMCs**

**Figure 6:**
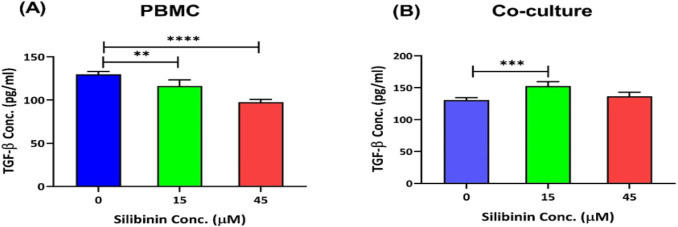
Silibin effect on the TGF-β levels of T cells in PBMCs and in the coculture condition. The effect of silibinin was determined on the TGF-β levels of T cells in PBMCs (A), and co-culture condition (B) (n=4). The graphs illustrate the mean±SD. Asterisks show significant changes as compared to non-treated cells (*p<0.05; **p<0.01; ***p<0.001; and ****p<0.0001).

The levels of TGF-β were significantly declined in PBMCs treated with silibinin, especially with 45 µM concentration (p=0.0068 with 15 µM, and p<0.0001 with 45 µM; Figure 6a). However, only 15 µM silibinin, increased TGF-β levels in the co-culture condition (p=0.0007, Figure 6b). 

## Discussion

Silibinin, a traditional Chinese medication, has been widely used for its antioxidant and anti-inflammatory characteristics; moreover, some evidence shows its favorable effects on cancer treatment (Tuki et al., 2021). In this *in vitro* study, we showed no adverse impact of silibinin on the PBMCs proliferation. Moreover, we found a protective role for silibinin on the proliferative capacity of PBMCs against the suppressive potential of the U-87 MG cells. Some previous studies have mentioned that silibinin stimulated the proliferation of lymphocytes. A study on rats undergoing partial hepatectomy (PHX) showed that treatment with silibinin and/or vitamin E prior to operation improved the lymphocyte proliferation as well as their cytokine secretion, which was attenuated in non-treated PHX animals (Horvath et al., 2001). Moreover, Johnson et al. (2003) observed a stimulatory effect of silymarin on phytohemagglutinin (PHA)-induced T cell proliferation only at low-dose (10 mg/kg) but not at higher doses (50 and 250 mg/kg) (Johnson et al., 2003). However, some others reported inconsistent results; for example, Dupuis et al. (2018) reported that silibinin exposure (50 μM) for 48 hr caused a reduction in both the proliferation and the production of the pro-inflammatory cytokines in T cells obtained from healthy donors as well as patients with active arthritis rheumatoid (Dupuis et al., 2018). In addition, another study pointed that silymarin suppressed both the proliferation and cytokine production in T cells; this study used three concentrations of silymarin (10, 50, and 100 μM), and the suppressive effect was observed only at 100 μM, but the other two concentrations induced no significant change on the T-cell proliferation, implying that 100 μM silibinin might be toxic for T-cells (Gharagozloo et al., 2011). Collectively, these data delineated that the effects of silibinin could be partially different depending on the dose and the condition used. Compelling evidence indicates no significant effect for silibinin (50 μM) on the proliferation of various cancer cells (Zeng et al., 2011; Gholami et al., 2017). Similarly, our results showed no change in the proliferation of silibinin-treated U-87 cell line. 

In this study, we showed an increase in the antioxidant properties of silibinin-treated U-87 MG cells. Recent data proposed that oxidative damage and the resulting generation of ROS are considered important carcinogens. In this regard, SOD3 and glutathione peroxidase can act as the cellular protective system against ROS (Miranda et al., 2000). Previous evidence disclosed that silibinin might exert its anti-cancer properties by altering the oxidative stress markers in favor of cytoprotective enzymes. In this context, Khan et al. (2014) observed that topical administration of silibinin in Swiss Albino Mice impairs cutaneous lipid peroxidation, an essential indicator of oxidative stress, and restores the activity of detoxifying enzymes in the skin tissue of the chemically induced skin carcinogenesis (Khan et al., 2014). Another *in vivo* study aimed to assess the silibinin effect on antioxidant activity in a colon cancer model; the results indicated that silibinin supplementation notably restored 1, 2 dimethylhydrazine (DMH)-induced decline in the antioxidant enzymes of the colon tissues. This group suggested a potent chemopreventive property for silibinin (Sangeetha et al., 2010). In contrast, Wang et al. (2020) reported an accumulation of free radicals, including superoxide, in silibinin-treated glioma cells, resulting in cell death; this study incubated cell line for two hours with 200 μmol/L silibinin, which was noticeably higher than the concentration that we selected for our research (Wang et al., 2020). 

In addition, we showed an increment in TAC levels and SOD3 activity in silibinin-treated PBMCs and in the co-culture condition. Previous studies indicated that the accumulation of ROS in T cells can cause functional impairment by suppressing the production of TH1-related cytokines (IL-2, IFN-γ, and TNF-α) (Aboelella et al., 2021; Huang et al., 2022). Malmberg et al. (2001) observed a reduction in TH1-related cytokines after exposure to exogenous H_2_O_2_. This group suggested that uncontrolled ROS generation in the tumor area might decrease the function of T cells (Malmberg et al., 2001). Another study designed tumor-specific chimeric Ag receptor (CAR) T cells co-expressing catalase (CAR-CAT) T cells to protect T cells against both intrinsic and extrinsic ROS and reported more vigorous cytotoxic activity of CAR-CAT T cells than CAR T cells under excess ROS production state in the tumor tissue. This study concluded that targeting ROS could sustain the tumor-killing ability of T cells in the TME and boost immunotherapy results (Ligtenberg et al., 2016).

IFNγ is a critical cytokine for antitumor response, mainly by supporting the activity of CTLs (Tu et al. 2021). The upregulation of this cytokine is considered a signature of CTL effector function (Abd Hamid et al., 2020; Han et al., 2023). Our data revealed a rise in the IFN-γ levels in the PBMCs from healthy donors treated with two different concentrations of silibinin. Similarly, Wu et al. (2021) elucidated the effect of silibinin on the tumor immune microenvironment and showed that the administration of liposomal silybin shifted the TME from a “cold” into a “hot” tumor via an increment in the infiltration of CTLs; also, liposomal silybin enhanced IFN-γ but diminished TGF-β levels. This group suggested that combining silibinin with other T-cell-stimulating therapies might be favorable for cancer targeting (Wu et al., 2021). Moreover, pretreatment with 200 or 400 mg/kg silibinin increased IFN-γ production in splenocytes of ovalbumin-sensitized BALB/c mice. Also, IgE levels were significantly diminished in silibinin-treated animals, supporting the role of silibinin in Th1 polarization (Kuo and Jan, 2009). On the other hand, Tyagi et al. (2012) pointed out that pretreatment with silibinin inhibits TNF-α and IFN-γ induced signaling in LM2 cell line (a suitable cell line for studying breast cancer metastasis). This study explained that IFN-γ could either promote or suppress tumor growth. In the context of cancer, excessive amounts of IFN-γ elicit the expression of cyclooxygenase-2 (COX-2) and inducible nitric oxide synthase (iNOS), both contributing to tumor progression (Tyagi et al., 2012). Therefore, using agents like silibinin to modulate the levels of IFN-γ is of utmost importance in cancer conditions.

Considering the decline in the suppressive effects of U-87 cells on T-cell proliferation and the protection of T cells following exposure to silibinin, we hypothesized that silibinin would improve T-cell proliferation in the coculture condition by lowering TGF-β levels in glioblastoma cell line. According to the literature on tumor cells and cancer models, TGF-β1 directly associates with tumor development and metastasis, mainly by promoting regulatory T cells (Wu et al., 2021; Fernandes et al., 2015). There is some evidence pointing to the inhibitory effect of silibinin on the production of TGF-β1 from tumor cells. For example, Li et al. (2018) reported that silibinin restrained TGF-β1-induced metastasis in renal transitional cell carcinoma (TCC) by downregulating the expression of COX-2 and limiting migration and progression of tumor cells. This research indicated a potential anti-metastatic role for silibinin in the future treatments of metastatic TCC (Li et al., 2018). Accordingly, we observed that both concentrations of silibinin lowered the levels of TGF-β in the treated PBMCs. However, we observed elevated levels of TGF-β in the co-culture condition treated only with the lower concentration of silibinin (15 μM). There are three isoforms of TGF-β, and according to available data, different treatments might affect the expression of one or more TGF-β isoforms (Prud'Homme 2007). For example, Kim et al. (2016) showed that silibinin treatment attenuated the expression of TGF-β2 and TGF-β-mediated metastasis in triple-negative breast cancer cells. However, this group observed no significant change in the levels of the TGF-β1 isoform (Kim et al., 2016). Furthermore, another study indicated that oral supplementation of Tranilast (analog of a metabolite of tryptophan) suppressed the production of TGF-β2 but not TGF-β1 in the experimental glioma model (Prud'Homme 2007; Platten et al., 2001). However, our data showed no significant effect for 45 µM silibinin on TGF-β production, and at 15 µM concentration, an increase was observed in the production of TGF-β in the coculture condition. Therefore, although this study demonstrated the protective ability of silibinin, more research is needed to discover the mechanisms involved in this ability.

To conclude, the present study provides evidence of the antioxidant properties of silibinin in tumor conditions. Interestingly, silibinin possesses partially protective effects on PBMCs against the suppressive effects of the U-87 MG cells. Furthermore, higher IFN-γ and lower TGF-β levels observed in silibinin-treated PBMCs could strengthen the anti-cancer potential of this medication.

## Conflicts of interest

The authors have declared that there is no conflict of interest.

## References

[B1] Abd Hamid M, Yao X, Waugh C, Rosendo-Machado S, Li S, Rostron T, Frankland J, Peng Y, Dong T (2020). Defective interferon gamma production by tumor-specific CD8+ T cells is associated with 5′ Methylcytosine-guanine Hypermethylation of interferon gamma promoter. Front Immunol.

[B2] Abenavoli L, Izzo AA, Milić N, Cicala C, Santini A, Capasso R (2018). Milk thistle (Silybum marianum): A concise overview on its chemistry, pharmacological, and nutraceutical uses in liver diseases. Phytother Res.

[B3] Aboelella NS, Brandle C, Kim T, Ding ZC, Zhou G (2021). Oxidative stress in the tumor microenvironment and its relevance to cancer immunotherapy. Cancers (Basel).

[B4] AbouZid S, Ahmed OM, Atta ur R (2013). Chapter 14 - Silymarin Flavonolignans: Structure–Activity Relationship and Biosynthesis. Studies in Natural Products Chemistry.

[B5] Afshari AR, Roshan MK, Soukhtanloo M, Ghorbani A, Rahmani F, Jalili-Nik M, Vahedi MM, Hoseini A, Sadeghnia HR, Mollazadeh H (2019). Cytotoxic effects of auraptene against a human malignant glioblastoma cell line. Avicenna J Phytomed.

[B6] Chen X, Song M, Zhang B, Zhang Y (2016). Reactive oxygen species regulate T cell immune response in the tumor microenvironment. Oxid Med Cell Longev.

[B7] Dias AS, Helguero L, Almeida CR, Duarte IF (2021). Natural compounds as metabolic modulators of the tumor microenvironment. Molecules.

[B8] Dupuis ML, Conti F, Maselli A, Pagano MT, Ruggieri A, Anticoli S, Fragale A, Gabriele L, Gagliardi MC, Sanchez M (2018). The natural agonist of estrogen receptor β silibinin plays an immunosuppressive role representing a potential therapeutic tool in rheumatoid arthritis. Front immunol.

[B9] Fernandes JV, Cobucci RNO, Jatobá CAN, de Medeiros Fernandes TAA, de Azevedo JWV, de Araújo JMG (2015). The role of the mediators of inflammation in cancer development. Pathol Oncol Res.

[B10] Ferry I, Kuzan-Fischer CM, Ernoult E, Rutka JT (2018). Targeting Cell Cycle Proteins in Brain Cancer. Handbook of Brain Tumor Chemotherapy, Molecular Therapeutics, and Immunotherapy.

[B11] Gao Y, Theng SS, Mah W-C, Lee CG (2015). Silibinin down-regulates FAT10 and modulate TNF-α/IFN-γ-induced chromosomal instability and apoptosis sensitivity. Biol Open.

[B12] Gharagozloo M, Jafari S, Esmaeil N, Javid EN, Bagherpour B, Rezaei A (2013). Immunosuppressive effect of silymarin on mitogen‐activated protein kinase signalling pathway: The impact on T cell proliferation and cytokine production. Basic Clin Pharmacol Toxicol.

[B13] Gholami M, Moallem SA, Afshar M, Etemad L, Karimi G (2017). Gestational exposure to silymarin increases susceptibility of BALB/c mice fetuses to apoptosis. Avicenna J Med Biotechnol.

[B14] Grabowski MM, Sankey EW, Ryan KJ, Chongsathidkiet P, Lorrey SJ, Wilkinson DS, Fecci PE (2021). Immune suppression in gliomas. J Neuro-oncol.

[B15] Han J, Wu M, Liu Z (2023). Dysregulation in IFN-γ signaling and response: the barricade to tumor immunotherapy. Front Immunol.

[B16] Hanif F, Muzaffar K, Perveen K, Malhi SM, Simjee Sh U (2017). Glioblastoma Multiforme: A review of its epidemiology and pathogenesis through clinical presentation and treatment. Asian Pac J Cancer Prev.

[B17] Horváth MÉ, Gonzalez-Cabello R, Blázovics A, van der Looij M, Barta I, Müzes G, Gergely P, Fehér J (2001). Effect of silibinin and vitamin E on restoration of cellular immune response after partial hepatectomy. J ethnopharmacol.

[B18] Hosseinabadi T, Lorigooini Z, Tabarzad M, Salehi B, Rodrigues CF, Martins N, Sharifi-Rad J (2019). Silymarin antiproliferative and apoptotic effects: Insights into its clinical impact in various types of cancer. Phytother Res.

[B19] Huang Y, Si X, Shao M, Teng X, Xiao G, Huang H (2022). Rewiring mitochondrial metabolism to counteract exhaustion of CAR-T cells. J Hematol Oncol.

[B20] Iraji F, Makhmalzadeh BS, Abedini M, Aghaei A, Siahpoush A (2022). Effect of herbal cream containing Fumaria officinalis and silymarin for treatment of eczema: A randomized double-blind controlled clinical trial. Avicenna J Phytomed.

[B21] Jafarzadeh A, Ahangar-Parvin R, Nemat M, Taghipour Z, Shamsizadeh A, Ayoobi F, Hassan ZM (2017). Ginger extract modulates the expression of IL-12 and TGF-β in the central nervous system and serum of mice with experimental autoimmune encephalomyelitis. Avicenna J Phytomed.

[B22] Jeong JC, Shin WY, Kim TH, Kwon CH, Kim JH, Kim YK, Kim KH (2011). Silibinin induces apoptosis via calpain-dependent AIF nuclear translocation in U87MG human glioma cell death. J Exp Clin Cancer Res.

[B23] Jiang K, Wang W, Jin X, Wang Z, Ji Z, Meng G (2015). Silibinin, a natural flavonoid, induces autophagy via ROS-dependent mitochondrial dysfunction and loss of ATP involving BNIP3 in human MCF7 breast cancer cells. Oncol Rep.

[B24] Johnson VJ, He Q, Osuchowski MF, Sharma RP (2003). Physiological responses of a natural antioxidant flavonoid mixture, silymarin, in BALB/c mice. Planta med.

[B25] Jung HJ, Park JW, Lee JS, Lee SR, Jang BC, Suh SI, Suh MH, Baek WK (2009). Silibinin inhibits expression of HIF-1alpha through suppression of protein translation in prostate cancer cells. Biochem Biophys Res Commun.

[B26] Khalili A, Fallah P, Hashemi SA, Ahmadian-Attari MM, Jamshidi V, Mazloom R, Beikzadeh L, Bayat G (2021). New mechanistic insights into hepatoprotective activity of milk thistle and chicory quantified extract: The role of hepatic Farnesoid-X activated receptors. Avicenna J Phytomed.

[B27] Khan AQ, Khan R, Tahir M, Rehman MU, Lateef A, Ali F, Hamiza OO, Hasan SK, Sultana S (2014). Silibinin inhibits tumor promotional triggers and tumorigenesis against chemically induced two-stage skin carcinogenesis in Swiss albino mice: possible role of oxidative stress and inflammation. Nutr Cancer.

[B28] Kim S, Han J, Jeon M, You D, Lee J, Kim HJ, Bae S, Nam SJ, Lee JE (2016). Silibinin inhibits triple negative breast cancer cell motility by suppressing TGF-β2 expression. Tumor Biol.

[B29] Kuo F-H, Jan T-R (2009). Silibinin attenuates antigen-specific IgE production through the modulation of Th1/Th2 balance in ovalbumin-sensitized BALB/c mice. Phytomedicine.

[B30] Li F, Sun Y, Jia J, Yang C, Tang X, Jin B, Wang K, Guo P, Ma Z, Chen Y (2018). Silibinin attenuates TGF‑β1‑induced migration and invasion via EMT suppression and is associated with COX‑2 downregulation in bladder transitional cell carcinoma. Oncol Rep.

[B31] Ligtenberg MA, Mougiakakos D, Mukhopadhyay M, Witt K, Lladser A, Chmielewski M, Riet T, Abken H, Kiessling R (2016). Coexpressed catalase protects chimeric antigen receptor–redirected T cells as well as bystander cells from oxidative stress–induced loss of antitumor activity. J Immunol.

[B32] Malmberg KJ, Arulampalam V, Ichihara F, Petersson M, Seki K, Andersson T, Lenkei R, Masucci G, Pettersson S, Kiessling R (2001). Inhibition of activated/memory (CD45RO+) T cells by oxidative stress associated with block of NF-κB activation. J Immunol.

[B33] Miranda A, Janssen L, Bosman CB, Van Duijn W, Oostendorp-van de Ruit MM, Kubben FJ, Griffioen G, Lamers CB, Han J, Van Krieken J (2000). Superoxide dismutases in gastric and esophageal cancer and the prognostic impact in gastric cancer. Clin Cancer Res.

[B34] Platten M, Wild‐Bode C, Wick W, Leitlein J, Dichgans J, Weller M (2001). N‐[3, 4‐dimethoxycinnamoyl]‐anthranilic acid (tranilast) inhibits transforming growth factor‐β release and reduces migration and invasiveness of human malignant glioma cells. Int J cancer.

[B35] Prud'Homme GJ (2007). Pathobiology of transforming growth factor β in cancer, fibrosis and immunologic disease, and therapeutic considerations. Lab Invest.

[B36] Ranjbar N, Saravani R, Faezizadeh Z (2020). Silymarin inhibits Toll-like receptor 8 gene expression and apoptosis in Ramos cancer cell line. Avicenna J Phytomed.

[B37] Sangeetha N, Aranganathan S, Nalini N (2010). Silibinin ameliorates oxidative stress induced aberrant crypt foci and lipid peroxidation in 1, 2 dimethylhydrazine induced rat colon cancer. Invest New Drugs.

[B38] Thomas DA, Massagué J (2005). TGF-β directly targets cytotoxic T cell functions during tumor evasion of immune surveillance. Cancer Cell.

[B39] Tu S, Lin X, Qiu J, Zhou J, Wang |H, Hu S, Yao Y, Wang Y, Deng Y, Zhou Y (2021). Crosstalk between tumor-associated microglia/macrophages and CD8-positive T cells plays a key role in glioblastoma. Front Immunol.

[B40] Tuli HS, Mittal S, Aggarwal D, Parashar G, Parashar NC, Upadhyay SK, Barwal TS, Jain A, Kaur G, Savla R (2021). Path of Silibinin from diet to medicine: A dietary polyphenolic flavonoid having potential anti-cancer therapeutic significance. Semin Cancer Biol.

[B41] Tyagi A, Agarwal C, Dwyer‐Nield LD, Singh RP, Malkinson AM, Agarwal R (2012). Silibinin modulates TNF‐α and IFN‐γ mediated signaling to regulate COX2 and iNOS expression in tumorigenic mouse lung epithelial LM2 cells. Mol Carcinog.

[B42] Wang C, He C, Lu S, Wang X, Wang L, Liang S, Wang X, Piao M, Cui J, Chi G (2020). Autophagy activated by silibinin contributes to glioma cell death via induction of oxidative stress-mediated BNIP3-dependent nuclear translocation of AIF. Cell Death Dis.

[B43] Weenink B, French PJ, Sillevis Smitt PAE, Debets R, Geurts M (2020). Immunotherapy in glioblastoma: Current shortcomings and future perspectives. Cancers (Basel).

[B44] Woroniecka KI, Rhodin KE, Chongsathidkiet P, Keith KA, Fecci PE (2018). T-cell dysfunction in glioblastoma: Applying a new framework T-cell dysfunction in glioblastoma. Clin Cancer Res.

[B45] Wu K-j, Zeng J, Zhu G-d, Zhang L-l, Zhang D, Li L, Fan J-h, Wang X-y, He D-l (2009). Silibinin inhibits prostate cancer invasion, motility and migration by suppressing vimentin and MMP-2 expression. Acta Pharmacol Sin.

[B46] Wu S, Liu D, Li W, Song B, Chen C, Chen D, Hu H (2021). Enhancing TNBC Chemo-immunotherapy via combination reprogramming tumor immune microenvironment with Immunogenic Cell Death. Int J Pharm.

[B47] Zeng J, Sun Y, Wu K, Li L, Zhang G, Yang Z, Wang Z, Zhang D, Xue Y, Chen Y (2011). Chemopreventive and chemotherapeutic effects of intravesical silibinin against bladder cancer by acting on mitochondria. Mol Cancer Ther.

